# A Randomized Controlled Trial of Allopurinol in Patients With Peripheral Arterial Disease

**DOI:** 10.1016/j.cjca.2015.05.010

**Published:** 2016-02

**Authors:** Alan J. Robertson, Allan D. Struthers

**Affiliations:** Division of Cardiovascular and Diabetes Medicine, University of Dundee, Ninewells Hospital and Medical School, Dundee, United Kingdom

## Abstract

**Background:**

Patients with peripheral arterial disease (PAD) are limited by intermittent claudication in the distance they can walk. Allopurinol has been shown in coronary arterial disease to prolong exercise before angina occurs, likely by prevention of oxygen wastage in tissues and reduction of harmful oxidative stress.

**Methods:**

In this study we evaluated whether allopurinol could prolong the time to development of leg pain in participants with PAD. In a double-blind, randomized controlled clinical trial participants were randomized to receive either allopurinol 300 mg twice daily or placebo for 6 months. The primary outcome was change in exercise capacity on treadmill testing at 6 months. Secondary outcomes were 6-minute walking distance, Walking Impairment Questionnaire, SF-36 questionnaire, flow-mediated dilatation, and oxidized low-density lipoprotein. Outcome measures were repeated midstudy and at the end of study. The mean age of the 50 participants was 68.4 ± 1.2 years with 39 of 50 (78%) male.

**Results:**

Five participants withdrew during the study (2 active, 3 placebo). There was a significant reduction in uric acid levels in those who received active treatment of 52.1% (*P* < 0.001), but no significant change in either the pain-free or the maximum walking distance. Other measures of exercise capacity, blood vessel function, and the participants' own assessment of their health and walking ability also did not change during the course of the study.

**Conclusions:**

Although allopurinol has been shown to be of benefit in a number of other diseases, in this study there was no evidence of any improvement after treatment in patients with PAD.

## Résumé

**Introduction:**

Les patients atteints d’une maladie artérielle périphérique (MAP) sont limités par une claudication intermittente dans la distance qu’ils peuvent parcourir. Il a été démontré que lors de maladie coronarienne l’allopurinol prolonge la période d’exercice avant que n’apparaisse l’angine, probablement par la prévention des pertes d’oxygène dans les tissus et la réduction des effets néfastes du stress oxydatif.

**Méthodes:**

Dans cette étude, nous avons évalué si l’allopurinol pourrait prolonger la durée avant la manifestation de la douleur à la jambe chez les participants atteints de MAP. Les participants d’un essai clinique à répartition aléatoire et à double insu étaient répartis de manière aléatoire pour recevoir soit l’allopurinol à raison de 300 mg 2 fois par jour ou le placébo durant 6 mois. Le principal critère de jugement était la modification de la capacité d’effort à l’épreuve de marche sur tapis roulant à 6 mois. Les critères secondaires étaient la distance de marche en 6 minutes, le Walking Impairment Questionnaire, le questionnaire SF-36, la dilatation médiée par le flux et les oxydés lipoprotéines de faible densité. Les mesures des résultats étaient répétées à la mi-étude et à la fin de l’étude. Cinquante participants dont 39 (78 %) étaient des hommes avaient un âge moyen de 68,4 ± 1,2 ans.

**Résultats:**

Cinq participants se sont retirés durant l’étude (2 du groupe sous traitement actif, 3 du groupe sous placébo). Une réduction significative des concentrations en acide urique de 52,1 % (*P* < 0,001) a été observée chez ceux qui recevaient le traitement actif, mais aucun changement significatif n’a été observé dans la distance de marche sans douleur ou maximale. Les autres mesures de la capacité d’effort, le fonctionnement des vaisseaux sanguins et la propre évaluation des participants concernant leur santé et leur habileté à la marche n’ont également pas changé au cours de l’étude.

**Conclusions:**

Bien qu’il ait été démontré que l’allopurinol est bénéfique contre plusieurs autres maladies, cette étude n’a montré aucune preuve d’amélioration après le traitement chez les patients atteints de MAP.

Peripheral arterial disease (PAD) is a disease that carries with it significant morbidity and through links with cardiovascular disease, mortality. The total disease prevalence has been estimated at 3%-10%, increasing with age to 15%-20% in those older than 70 years of age.^1^, ^2^ However, much of this disease is asymptomatic, as demonstrated by the Edinburgh Artery Study.^3^ Patients with major asymptomatic disease had a 60% increase in relative risk of cardiovascular disease.^3^ The cornerstone of treatment of PAD therefore remains identification and treatment of cardiovascular risk factors, however, from a patient perspective the most troubling problem remains that of intermittent claudication.^4^

An inevitable consequence of normal intracellular metabolism is the production of reactive oxygen species.^5^ Of particular relevance is the production of hydrogen peroxide and superoxide. This happens as a by-product of the formation of uric acid by xanthine oxidoreductase (XOR) and thus blockade of this process might be beneficial in reducing the endothelial dysfunction caused by reactive oxygen species. Allopurinol is a well-established drug that is highly effective in blocking XOR and reducing uric acid levels. It is therefore a mainstay of the preventive treatment of gout but has shown some promise in a number of cardiovascular conditions; most recently de Abajo et al.^6^ showed in a case-control study that allopurinol use (in particular at higher doses and for longer periods) was associated with a lower risk of non-fatal myocardial infarction.^6^, ^7^, ^8^, ^9^, ^10^ However, the most striking symptomatic effect has been that demonstrated by Noman et al. in patients with angina: their study of 65 patients with angiographically documented coronary artery disease, a positive exercise tolerance test, and stable chronic angina pectoris demonstrated an almost one-fifth improvement in time to ST depression, time to development of chest pain, and total exercise time.^9^

This antianginal effect raises questions about its potential mechanism. One of these could be that oxygen is a substrate for XOR and hence XOR blockade with allopurinol might boost tissue oxygen and thus promote an oxygen-sparing effect. If this were the case, one would postulate that allopurinol might exert an anti-ischemic effect in PAD. However, the other possible mechanism of this anti-anginal effect is that of an offloading effect on the heart. It is known that patients with angina benefit from drugs that reduce afterload on the heart, because this decreases the oxygen demand of the heart, thereby improving the oxygen supply/demand ratio. Rekhraj et al. showed that allopurinol was capable of achieving this offloading effect by reducing end-systolic volume, however, if this is the primary mechanism of action it would not apply in PAD and hence there would be no benefit in PAD.^8^ We conducted this study to help answer this mechanistic question.

The aim of this randomized controlled trial was to examine whether high-dose allopurinol prolongs exercise duration in patients with PAD.

## Methods

This study was a randomized, double-blind parallel-group placebo-controlled clinical trial, run in accordance with the Declaration of Helsinki and The Medicines for Human Use (Clinical Trials) (Amendment) Regulations, 2006. Approvals were received from local National Health Service (NHS) Research and Development, Scotland A Research Ethics Committee, University of Dundee/NHS Tayside (as cosponsor), and the Medicines and Healthcare Products Regulatory Agency (MHRA). The study was registered with the International Standard Randomised Controlled Trial Number (ISRCTN01772998), ClinicalTrials.gov (NCT01147705), and EudraCT (2010-020662-23).

Patients were recruited after their attendance at the Intermittent Claudication Clinic at Ninewells Hospital, Dundee. Where patients met the inclusion/exclusion criteria (Table 1) on the basis of their clinic letter a covering letter was sent out from the clinician they had seen in clinic, enclosing a Participant Information Sheet and a prepaid reply slip. All those who replied and indicated they would be interested in participating in the study were telephoned by A.J.R. Missing information relevant to inclusion/exclusion criteria was checked in this phone call and then patients were offered an appointment for an initial screening visit.

All study visits were conducted at the Department of Clinical Pharmacology, Ninewells Hospital, Dundee, between March 2011 and June 2012. After written informed consent, an initial clinical assessment was undertaken before baseline exercise testing and phlebotomy. At the second visit a repeat treadmill test was carried out to check for reproducibility—if variance was < 25% then baseline measurements of the outcome measures described herein were carried out and participants were provided with an initial supply of the investigational medicinal product (IMP).

Randomization had been carried out by Tayside Pharmaceuticals (Ninewells Hospital, Dundee) as supplier of the IMP, before commencement of study visits. This was achieved via a computer-generated randomization system using 5 blocks of 10 to ensure equal numbers between active and placebo groups. The IMP supply was sequentially numbered and the randomization key held in sealed envelopes by Tayside Pharmaceuticals, Ninewells Pharmacy (in case of any need for emergency unblinding), and for safe-keeping in a locked fireproof cabinet by the Asthma & Allergy Research Group, Ninewells. The investigating team did not have access to the key until after analysis had taken place.

Participants were randomized to receive either allopurinol or placebo for the 6 months of participation. The initial dose was 100 mg once daily of allopurinol (or placebo) for 2 weeks, increased to 300 mg once daily for 4 weeks, then continued at 300 mg twice daily for the remainder of the study.

Exercise tolerance testing (ETT) was conducted using the Gardner protocol, as is common in PAD studies to test efficacy of new treatments.^11^, ^12^ The machine used was a GE Marquette Series 2000 Treadmill Stress Testing System (GE Healthcare Clinical Systems [UK] Ltd, Hatfield, UK). The tests were conducted in accordance with the University of Dundee standard operating procedures for ETT and were supervised by A.J.R. (Principal Investigator and Advanced Life Support instructor) and a second member of staff.

Six-minute walk tests (6MWTs) were carried out as per American Thoracic Society guidelines, in a quiet corridor on a 30-m track marked at 1-m intervals.^13^

Endothelial function was assessed by measuring flow-mediated dilatation (FMD) of the brachial artery. FMD was measured using a Sequoia 512 (Siemens, Camberley, UK) ultrasound machine with an 8-MHz linear array probe. Our standard departmental protocol was used to carry out the measurements, which is based on the International Brachial Artery Reactivity Task Force guidelines.^14^ The FMD was analyzed using a Brachial Analyzer for Research, part of the Medical Imaging Applications Vascular Research Tools software suite (version 5.6.12, Medical Imaging Applications LLC, Coralville, IA), again using our departmental protocol. The acquisition and analysis of the FMD images were performed by A.J.R., who was blinded to the allocated treatment.

Blinded analysis of oxidized low-density lipoprotein (Ox-LDL), a plasma marker of oxidative stress, was carried out by the Vascular and Inflammatory Diseases Research Unit in Ninewells Hospital, Dundee, using a Mercodia Oxidised-LDL enzyme-linked immunosorbent assay.

The Walking Impairment Questionnaire (WIQ) is a short and straightforward questionnaire widely used in the evaluation of PAD patient symptoms, which provides good correlation with patient performance on the treadmill, especially when patients are stratified into high and low performance groups.^15^, ^16^, ^17^, ^18^, ^19^ The SF-36 is a multipurpose, short-form health survey consisting of 36 questions that provide summary measures for functional health and well-being and a psychometrically-based physical and mental health summary.^20^

A meta-analysis of exercise treatment trials in PAD and several drug trials was initially used to guide the required power for the study. In randomized trials in PAD, the standard deviation (SD) tends to be approximately 30 metres.^21^, ^22^, ^23^, ^24^ In drug treatment trials, the time to claudication usually increases by approximately 30 metres (on average 30% more than placebo).^21^, ^22^, ^23^, ^24^ A 30-m improvement is thought to make a clinically significant difference in PAD. If it is assumed a 30-metre improvement with a 30-metre SD, then 42 subjects total in a parallel group study would be required to demonstrate a 30-metre improvement over placebo (for 90% power at *P* < 0.05). However to allow for dropouts, 50 PAD subjects were enrolled in this study.

Analysis was undertaken using IBM SPSS Statistics version 20 (IBM Corp, Armonk, NY). Data were checked for normality using the Shapiro-Wilk test (because of the smaller sample sizes involved in this study). Where the significance value of the Shapiro-Wilk test was < 0.05 the data were assumed to be nonnormally distributed. In these situations it was log-transformed to see if this led to a normal distribution, thus allowing parametric analysis to be carried out. If it remained nonnormally distributed then nonparametric analysis was used. Pearson χ^2^ was used for discrete variables. An independent samples *t* test was used for normally distributed continuous variables and a Mann-Whitney *U* test for nonparametric data. For repeated comparisons within the same subject, a paired *t* test was used for normally distributed data and a Wilcoxon signed-rank matched pairs test for nonparametric data. A 2-factor analysis of variance with repeated measures on 1 factor was also used for the primary end points.

The primary end point was the change in distance walked on an exercise tolerance test from baseline over a 6-month period. This comprised the claudication onset distance (COD: when the participant first noticed claudication pain) and the peak walking distance (PWD: when they could walk no further and had to terminate the treadmill test).

The overall secondary objectives were to: (1) see if allopurinol improved the quality of life in patients with PAD; and (2) to investigate the antioxidant effects of allopurinol in patients with PAD. To that end there were a number of secondary end points, namely the aforementioned 6MWT, WIQ/SF-36 questionnaires, FMD, and Ox-LDL.

## Results

**Figure 1:**
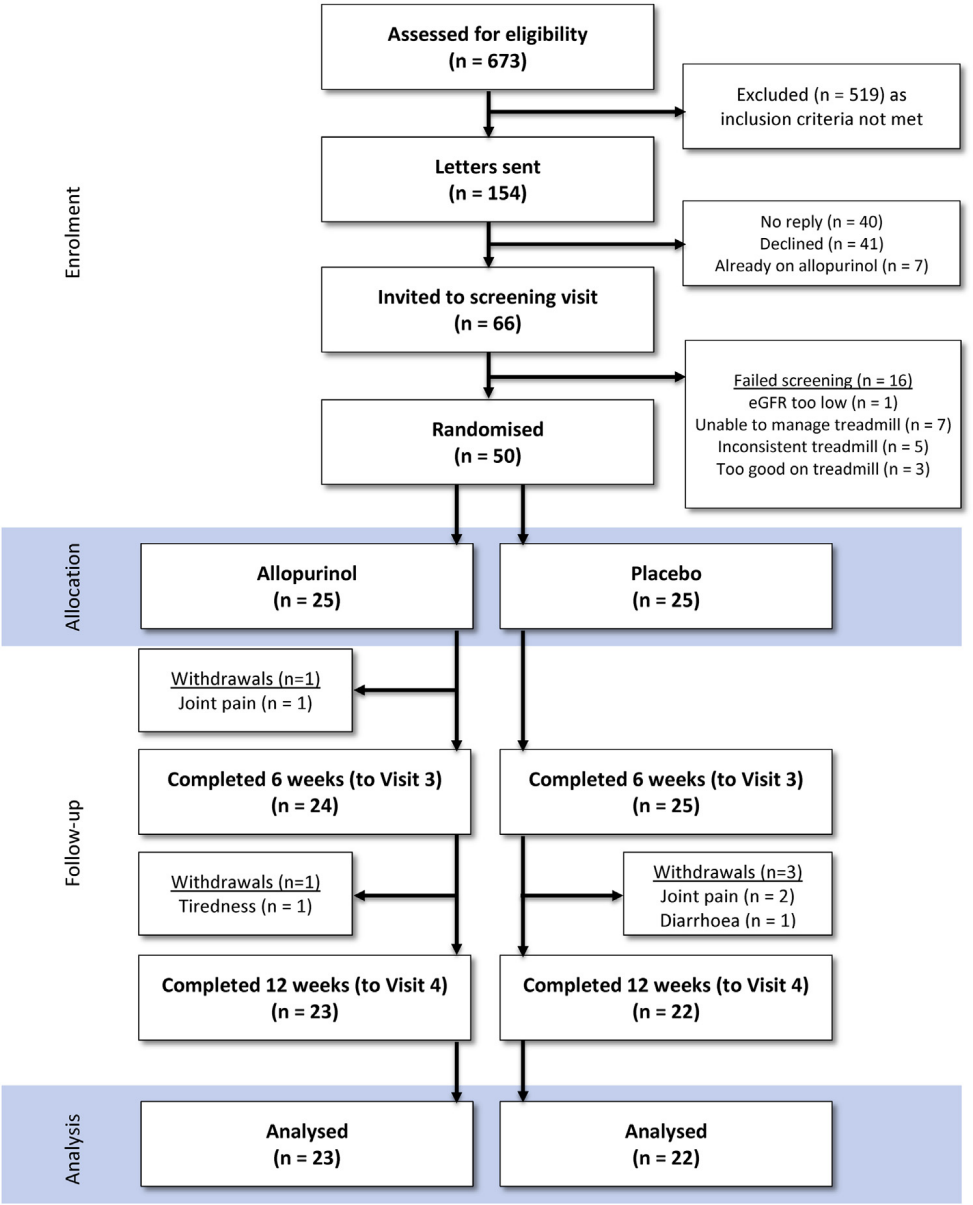
Consolidated Standards of Reporting Trials diagram.

In total, 50 patients were recruited, 5 withdrew during the course of the study (allopurinol, n = 2; placebo, n = 3; Fig. 1). Of the 50 patients randomized, 78% (39 of 50) were male. Participants had a mean age of 68.4 ± 1.2 years. The baseline demographic characteristics were as outlined in Table 2.

**Figure 2:**
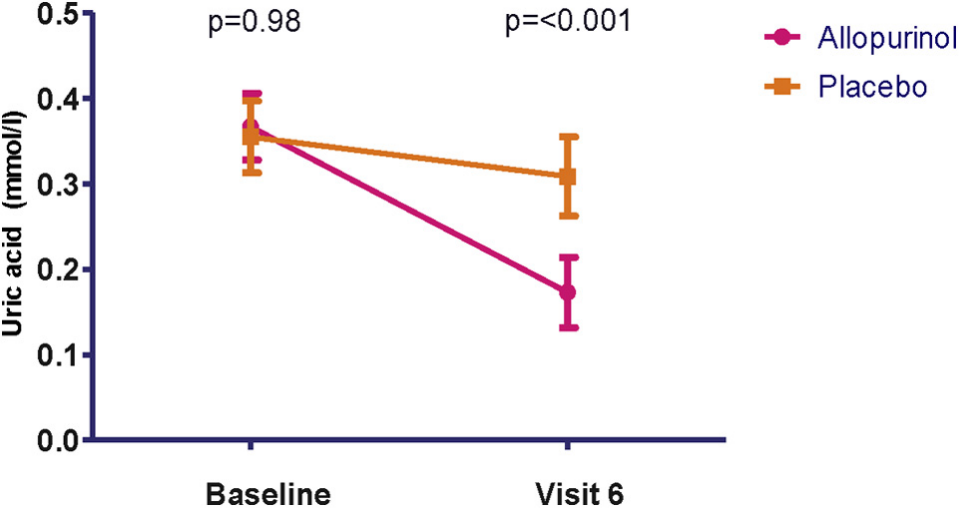
Reduction in uric acid (**vertical bars** indicate 95% confidence interval).

Uric acid levels were checked at the initial and final visits, which provided a good idea of concordance with treatment. As noted in Table 2, there was no significant difference in baseline uric acid levels between the 2 groups (*P* = 0.98). There was a highly significant decrease in uric acid for those who received treatment, with an order of magnitude of 52.1% (*P* < 0.001; Fig. 2). A small 10.0% decrease was noted in the placebo group but this was not statistically significant (*P* = 0.06). All other participants in the active treatment arm had > 13% reduction, with most a 40%-60% reduction as indicated by the aforementioned mean.

The baseline, midpoint, and end of study treadmill values for the COD and PWD are shown in Table 3. Two-factor analysis of variance analysis showed no significant difference between the 2 groups over time with *P* = 0.35 (COD) and *P* = 0.75 (PWD).

No significant difference in the 6MWT distances were noted between the 2 arms of the study, as shown in Table 4.

Baseline FMD measurements were similar in the 2 groups, with no significant changes noted over the course of treatment (Table 5).

Baseline WIQ scores were 132.9 (95% confidence interval [CI], 108.7-157.1) in the allopurinol group and 140.0 (95% CI, 111.1-168.8) in the placebo group (*P* = 0.92). By the end of the study these had not significantly changed, with values of 139.7 (95% CI, 96.2-183.2) and 146.1 (95% CI, 111.8-180.3), respectively (*P* = 0.34). Subdivision into the distance, speed, and climb elements showed similar results.

Baseline SF-36 scores were 535.1 (95% CI, 457.7-612.5) in the allopurinol group and 571.0 (95% CI, 519.5-622.4) in the placebo group (*P* = 0.73). Values at the final visit were 555.2 (95% CI, 485.0-625.5) and 536.5 (95% CI, 470.8-602.2), respectively (*P* = 0.57). Analysis of the various subdomains of SF-36 also showed no significant difference.

Baseline Ox-LDL values were 53.0 U/L in the allopurinol group (95% CI, 45.7-60.3) and 49.4 U/L (95% CI, 43.1-55.8) in the placebo group (*P* = 0.62). These did not significantly change during the course of the study.

There was 1 serious adverse event (community-acquired pneumonia requiring hospital admission). No significant changes in urea and electrolytes or liver function tests were noted in the course of the study in either group.

## Discussion

There was no significant difference in baseline COD and PWD measurements in the placebo group (despite a greater pack-year history in this group). By the end of the study both measures had increased in each arm of the study—this was on the order of 40 m for COD and 27 m for PWD, again with no significant difference between active and placebo groups. This temporal increase in walking distance in the placebo arm is something that has been recognized previously in studies of PAD.^25^ To investigate if there was any subgroup that showed benefit from allopurinol, the treadmill results were split into those with high/low baseline COD/PWD and those with high/low baseline uric acid/systolic blood pressure and ankle-brachial index (see Supplementary Material). None of these subdivisions changed the results and there remained no statistically significant difference between the 2 groups after 6 months of treatment.

In consideration of the FMD results, it can be seen that the baseline values were similar in the active and placebo groups. The relative change in vessel diameter after hyperemia (ie, after blood pressure cuff deflation) was very slightly better in the allopurinol group, however, the absolute level was still very small (< 0.5%) and the difference between the active and placebo groups was not statistically significant. Although these findings are in keeping with other outcome measures in this trial, a number of other studies of allopurinol have previously shown an improvement in FMD.^8^ In one other recent study of allopurinol by Szwejkowski et al. no effect on endothelial function was found. They postulated that the lack of effect on FMD results in their study might be because baseline FMD was high with little room for further improvement. This might be that their patients with type II diabetes mellitus were very well treated with statins, angiotensin-converting enzyme inhibitors, and angiotensin receptor blockers—all of which are known to improve endothelial function.^7^ Prescription of these agents was reassuringly high also in this population of PAD patients (see Table 2), however it also has to be considered that the patient population studied, all with proven PAD, had stiffer vessels that were less likely to respond to treatment.^26^, ^27^

We know that allopurinol has been shown to be of benefit in angina, in some ways a similar disease process, yet in this study it has not been of benefit in patients with PAD. Rekhraj et al showed that allopurinol reduced left ventricular (LV) end-systolic volume and LV afterload (measured using the augmentation index) in patients with ischemic heart disease, which suggests that offloading the left ventricle might be another mechanism that contributes to the anti-ischemic effect of allopurinol in angina pectoris.^8^ This particular effect on LV afterload would produce an anti-ischemic effect on the heart but not an anti-ischemic effect in PAD. Our negative finding in PAD therefore implies that the mechanism of action of allopurinol in angina is its LV offloading effect and not a direct oxygen-sparing effect by blocking an oxidase enzyme that “wastes” tissue oxygen.

With PAD a factor might also be a different calibre of vessel. The coronary arteries at their largest (the left main stem) measure 4.5 ± 0.5 mm, and decreases to 1.9 ± 0.4 mm in the distal left anterior descending artery.^28^ In comparison, the peripheral arteries in the legs are more than twice this diameter—with the common femoral artery having a diameter of 9.3 ± 1.1 mm, and decreases to 4.9 ± 0.6 mm when it reaches the distal popliteal artery.^29^ However, the extent to which this is a true comparison is unknown, because in the later stages of PAD much of the lower limb supply is via collateral vessels, which might in fact be of a smaller calibre than coronary vessels.

This study was based in a single centre, with a relatively small number of participants in absolute terms. Two participants had either no change or an increase in uric acid level between start and end of the study (which suggests at least a period of noncompliance), however, in general the IMP was well tolerated with only a small number of adverse events reported. There were issues in consistent assessment of the COD with treadmill testing because of the variation between participants in their reporting of pain—some would forget to mention it and only volunteer the information with prompting. There was also a clear heterogeneity in the participants with regard to severity of PAD—some managed to walk only 1 or 2 minutes on the treadmill before having to stop because of pain, yet others were able to manage a much more considerable distance. In some respects this could be seen as a limitation, however, in reality it reflects the clinical spectrum of PAD. Attempts were made in the analysis to subdivide the participants into ‘poor’ and ‘good’ walkers with regard to COD and PWD; it could be argued that this subdivision reduced the number available for analysis by too great a degree (the study was certainly not powered for such comparisons), and that greater number of participants (or a focus on particularly good or poor walkers) could have been beneficial, however, there did not appear to be any trend that would have reached statistical significance with a greater number of participants.

With regard to the 6MWT there is an element of a ‘learning effect’ at play. This is something that has been recognized previously and is believed to be on the order of approximately 4.5% in healthy subjects, possibly greater in those with more disease burden.^30^ It tends to decrease with repeated test administration, plateauing after 3 tests.^31^ In this study, 3 tests were carried out (baseline/mid/end). Only a small learning effect of approximately 3% appeared to be present but an increased number of tests would have helped to reduce the effect of this. This would, however, have to be balanced by the fatigue experienced by patients—additional walking tests would have either necessitated extra study visits or a much increased visit length to allow for sufficient rest, potentially reducing the acceptability of the study protocol to participants.

In conclusion, treatment with allopurinol did not prolong exercise duration in patients with PAD. All other measures of exercise, vascular health, and indeed the participants' own assessment of their health according to questionnaire responses did not change.

In view of the excellent reduction in uric acid levels in the allopurinol group this would suggest that it is the treatment itself that does not work in this particular disease process rather than a failure to properly block XOR. This study once again underscores that despite there being many effective antianginal therapies, in general these are of little benefit in PAD.

As highlighted at the beginning of this article, there were 2 potential mechanisms at play for how allopurinol has shown benefit in cardiac ischemia—either by reduction of oxygen wastage to the tissues or via offloading the heart. Because of the conclusively negative results in PAD presented herein, they suggest that LV offloading is the main mechanism underlying the antianginal effect of allopurinol.

## Figures and Tables

**Table 1:** Inclusion/exclusion criteria

Inclusion	• Men and women age 35-85 years of age who suffer from PAD • PAD defined as: ○ Claudication defined as leg pain with walking that disappears within 10 minutes with standing and of presumed atherosclerotic origin and ○ An ankle brachial pressure index of < 0.90 on the worst leg at rest • Stable disease demonstrated by a reproducible pain-free walking distance on 2 consecutive treadmill tests (ie, less than 25% variance) • The reason for termination of the test must be claudication pain only
Exclusion	• Rest pain • Childbearing potential without adequate contraceptive measures • Heart failure or any other exercise-limiting cardiac disease • Blood pressure > 180/100 mm Hg • Renal or liver disease • Malignancy • Already receiving allopurinol or had an adverse reaction to it • Recent marked change in symptoms or recent (in the past 6 months) intervention for PAD • Receiving treatment with either 6-mercaptopurine, azathioprine, warfarin, or theophylline

PAD, peripheral arterial disease.

**Table 2:** Characteristics of participants at baseline

	Allopurinol (n = 25)	Placebo (n = 25)	*P*
Mean age, years	69.6 (9.1)	67.3 (7.5)	0.34
Male sex, n (%)	21 (84)	18 (72)	0.50
Height, m	1.69 (0.09)	1.66 (0.07)	0.28
Weight, kg	77.7 (16.6)	80.6 (16.6)	0.36
BMI	27.2 (5.1)	29.1 (5.2)	0.21
Pulse, beats per minute	76.5 (11.6)	75.4 (14.9)	0.62
Pack-years	31.7 (21.0)	49.7 (37.0)	0.04
Systolic BP, mm Hg	153 (21)	156 (20)	0.65
Diastolic BP, mm Hg	76 (11)	79 (10)	0.27
ABI	0.61 (0.12)	0.60 (0.12)	0.65
Average weekly alcohol intake (units per week)	7.4 (10.0)	16.8 (18.8)	0.08
Smoking status			
Current smoker	6	7	0.75
Ex- or nonsmoker	19	18	
Uric acid, mmol/L	0.36 (0.09)	0.34 (0.09)	0.98
Concomitant medications, n (%)			
Aspirin/clopidogrel	24 (96)	23 (92)	0.88
Statin	23 (92)	24 (96)	0.88
ACEi/ARB	17 (68)	18 (72)	0.87

Data are presented as mean (SD) except where otherwise noted.

ABI, ankle-brachial index; ACEi, angiotensin-converting enzyme inhibitor; ARB, angiotensin receptor blocker; BMI, body mass index; BP, blood pressure.

**Table 3:** Treadmill walking distance in metres at different study stages

Time point	Allopurinol (95% CI)	Placebo (95% CI)	*P*
COD baseline	137.1 (82.0-192.2)	151.7 (105.4-198.0)	0.31
COD visit 4	161.7 (109.0-214.3)	155.0 (110.2-200.0)	0.95
COD visit 6	180.0 (111.5-248.6)	177.1 (133.1-221.2)	0.48
PWD baseline	315.5 (206.0-425.0)	362.6 (266.4-458.8)	0.24
PWD visit 4	320.1 (209.0-431.2)	341.5 (256.9-426.1)	0.42
PWD visit 6	347.4 (224.8-470.0)	358.4 (253.9-462.9)	0.61

CI, confidence interval; COD, claudication onset distance; PWD, peak walking distance.

**Table 4:** Six-minute walk test distance in metres at different study stages

Time point	Allopurinol (95% CI)	Placebo (95% CI)	*P*
Baseline	401.2 (371.7-430.7)	389.1 (369.2-409.1)	0.72
Visit 4	396.3 (366.1-426.6)	395.6 (376.3-414.8)	0.97
Visit 6	398.2 (374.4-422.1)	399.9 (375.8-424.0)	0.92
Mean change from baseline to Visit 4	4.4 (−10.5 to 19.4)	8.7 (−2.4-19.8)	0.73
Mean change from baseline to Visit 6	6.4 (−3.9 to 16.6)	13.1 (1.0-25.1)	0.63

CI, confidence interval.

**Table 5:** Flow-mediated dilatation: baseline vessel diameter and percentage change throughout study

Time point	Allopurinol (95% CI)	Placebo (95% CI)	*P*
Baseline vessel size prior to cuff inflation, mm	4.66 (4.34-4.98)	4.24 (3.75-4.73)	0.14
Absolute change post-cuff at visit 2 (initial visit), mm	0.38 (0.25-0.51)	0.34 (0.19-0.50)	0.65
Relative change post-cuff at visit 2 (initial visit), mm	8.33 (5.87-10.79)	9.61 (4.92-14.30)	0.84
Absolute change post-cuff at visit 5, mm	0.24 (0.16-0.31)	0.24 (0.13-0.34)	0.66
Relative change post-cuff at visit 5, %	4.85 (3.46-6.24)	5.61 (3.54-7.69)	0.32
Relative change compared with baseline post-cuff at visit 5, %	−0.29 (−0.53 to −0.06)	−0.15 (−0.59 to 0.28)	0.42
Absolute change post-cuff at visit 6, mm	0.41 (0.30-0.52)	0.25 (0.14-0.36)	0.06
Relative change post-cuff at visit 6, %	8.92 (6.43-11.40)	6.55 (3.49-9.61)	0.27
Relative change compared with baseline post-cuff at visit 6, %	0.41 (−0.30 to 1.11)	−0.28 (−0.64 to 0.09)	0.32

“Post-cuff” indicates the measurement of maximal vessel size that was made immediately following deflation of the blood pressure cuff.

CI, confidence interval.
