# Supplementary material for: A Randomized Controlled Trial of Allopurinol in Patients With Peripheral Arterial Disease
Source: Can J Cardiol. 2016 Feb;32(2):190–6. doi: 10.1016/j.cjca.2015.05.010 (PMC4742517; doi:10.1016/j.cjca.2015.05.010)
Supplement: Supplementary Material [file mmc1.pdf]

Subgroup Analysis of Exercise Test Results

1.1 Treadmill testing

The data was sub-divided by a variety of factors:

- Factor: above/below median baseline claudication onset (section 1.1.1)
- Factor: above/below median baseline peak walking distance (section 1.1.2)
- Factor: above/below median baseline uric acid (section 1.1.3)

- Factor: above/below median baseline systolic BP (section 0)
- Factor: above/below median baseline ABI (section 1.1.5)

1.1.1 Factor: above/below median baseline claudication onset

The median claudication onset distance at baseline was 98.4m. The participants were split into two groups – those below and those above this median level. The relative increases in both claudication onset distance and peak walking distance were then calculated.

		Allopurinol (95% CI)	Placebo (95% CI)	p
Relative increase in COD from Baseline to Visit 4	Below median baseline COD	0.55 (0.25 to 0.85)	0.29 (0.07 to 0.51)	0.45
	Above median baseline COD	0.11 (-0.13 to 0.36)	0.18 (-0.02 to 0.38)	0.70
Relative increase in COD from Baseline to Visit 6	Below median baseline COD	0.53 (0.13 to 0.93)	0.74 (0.38 to 1.09)	0.35
	Above median baseline COD	0.27 (-0.22 to 0.76)	0.27 (-0.07 to 0.60)	0.75

Supplemental Table S1 – Relative increase in COD split by low/high baseline COD

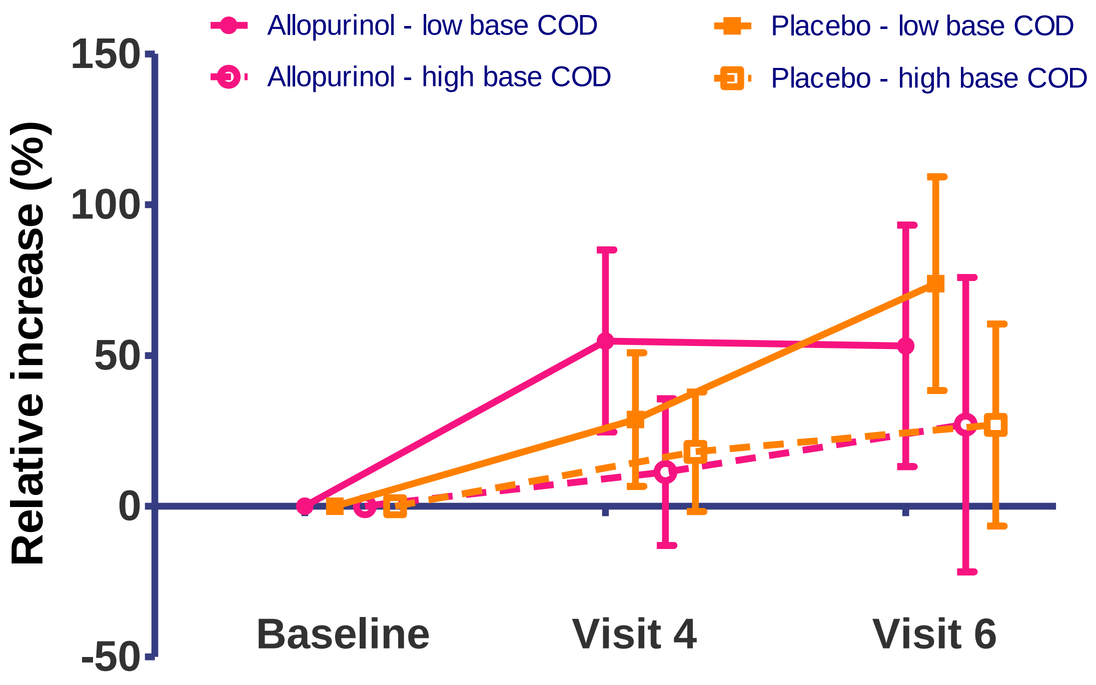

Supplemental Figure S1 – Relative increase in COD split by low/high baseline COD

		Allopurinol (95% CI)	Placebo (95% CI)	p
Relative increase in PWD from Baseline to Visit 4	Below median baseline COD	0.03 (-0.20 to 0.26)	0.29 (0.07 to 0.51)	0.45
	Above median baseline COD	0.00 (-0.14 to 0.14)	0.18 (-0.02 to 0.38)	0.75
Relative increase in PWD from Baseline to Visit 6	Below median baseline COD	0.24 (-0.14 to 0.63)	0.16 (-0.02 to 0.34)	0.74
	Above median baseline COD	0.02 (-0.12 to 0.17)	0.09 (-0.08 to 0.25)	0.48

Supplemental Table S2 – Relative increase in PWD split by low/high baseline COD

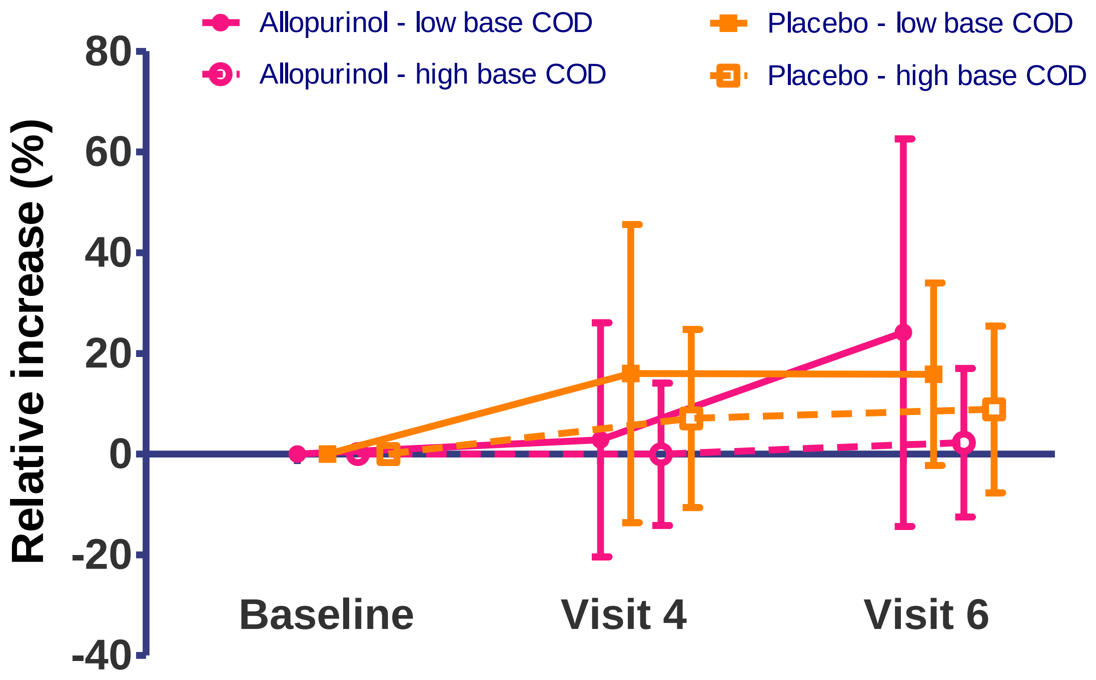

Supplemental Figure S2 – Relative increase in PWD split by low/high baseline COD

1.1.2 Factor: above/below median baseline peak walking distance

The median peak walking distance at baseline was 278.1m. The participants were split into two groups – those below and those above this median level. The relative increases in both claudication onset distance and peak walking distance were then calculated.

		Allopurinol (95% CI)	Placebo (95% CI)	p
Relative increase in COD from Baseline to Visit 4	Below median baseline PWD	0.49 (0.14 to 0.85)	0.20 (0.00 to 0.40)	0.41
	Above median baseline PWD	0.20 (-0.04 to 0.44)	0.28 (0.05 to 0.50)	0.82
Relative increase in COD from Baseline to Visit 6	Below median baseline PWD	0.37 (-0.10 to 0.84)	0.36 (0.03 to 0.69)	0.79
	Above median baseline PWD	0.44 (0.01 to 0.87)	0.70 (0.27 to 1.09)	0.23

Supplemental Table S3 – Relative increase in COD split by low/high baseline PWD

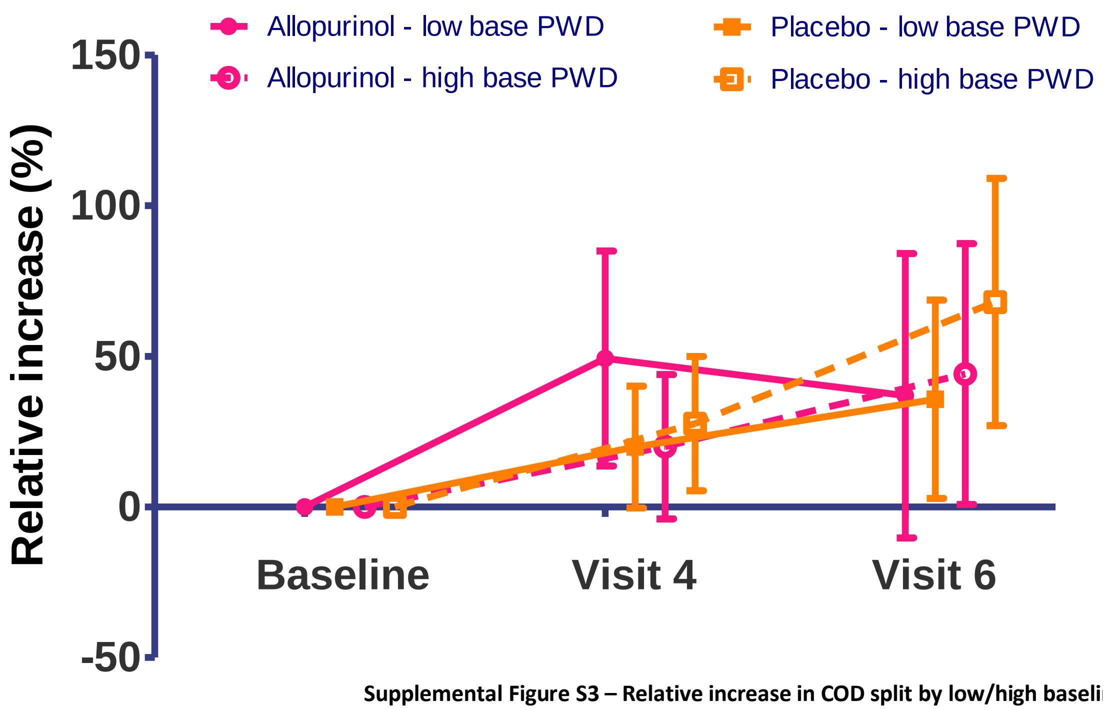

Supplemental Figure S3 – Relative increase in COD split by low/high baseline PWD

		**Allopurinol (95% CI)**	**Placebo (95% CI)**	**p**
Relative increase in PWD from Baseline to Visit 4	Below median baseline PWD	0.08 (-0.17 to 0.32)	0.15 (-0.14 to 0.44)	0.88
	Above median baseline PWD	-0.04 (-0.18 to 0.10)	0.07 (-0.08 to 0.23)	0.28
Relative increase in PWD from Baseline to Visit 6	Below median baseline PWD	0.24 (-0.19 to 0.67)	0.10 (-0.08 to 0.28)	0.93
	Above median baseline PWD	0.04 (-0.09 to 0.17)	0.16 (0.00 to 0.32)	0.23

Supplemental Table S4 – Relative increase in PWD split by low/high baseline PWD

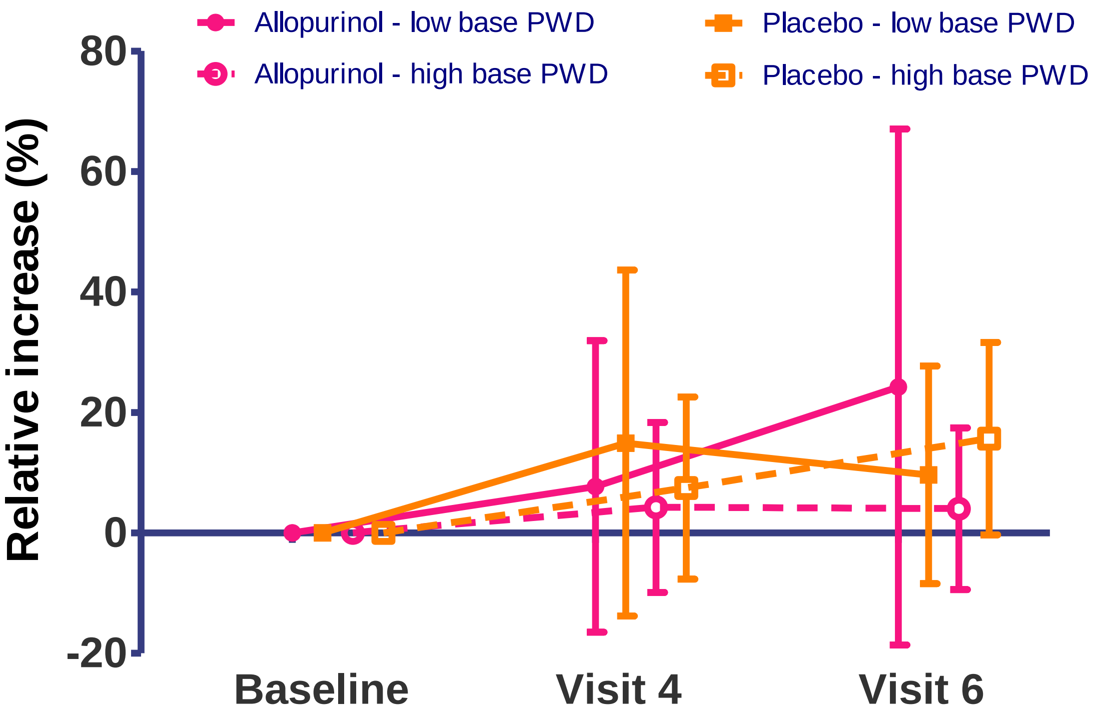

Supplemental Figure S4 – Relative increase in PWD split by low/high baseline PWD

1.1.3 Factor: above/below median baseline uric acid

The median uric acid at baseline was 0.36mmol/l. The participants were split into two groups – those below and those above this median level. The relative increases in both claudication onset distance and peak walking distance were then calculated.

		Allopurinol (95% CI)	Placebo (95% CI)	p
Relative increase in COD from Baseline to Visit 4	Below median baseline urate	0.37 (0.12 to 0.61)	0.17 (-0.05 to 0.38)	0.32
	Above median baseline urate	0.30 (-0.10 to 0.71)	0.28 (0.08 to 0.48)	0.78
Relative increase in COD from Baseline to Visit 6	Below median baseline urate	0.33 (0.06 to 0.60)	0.27 (-0.09 to 0.62)	0.85
	Above median baseline urate	0.51 (-0.15 to 1.16)	0.67 (0.32 to 1.01)	0.31

Supplemental Table S5 – Relative increase in COD split by low/high baseline urate

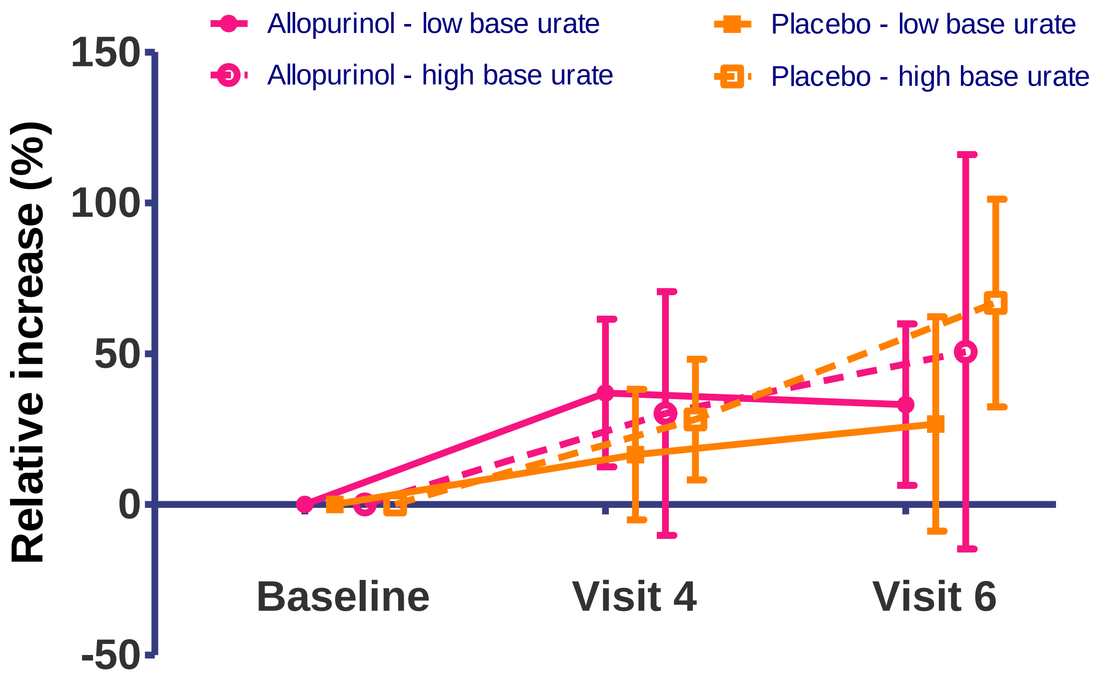

Supplemental Figure S5 – Relative increase in COD split by low/high baseline urate

		Allopurinol (95% CI)	Placebo (95% CI)	P
Relative increase in PWD from Baseline to Visit 4	Below median baseline urate	-0.13 (-0.25 to -0.01)	0.11 (-0.20 to 0.42)	0.11
	Above median baseline urate	0.21 (0.00 to 0.42)	0.12 (-0.08 to 0.33)	0.69
Relative increase in PWD from Baseline to Visit 6	Below median baseline urate	-0.02 (-0.14 to 0.09)	0.08 (-0.16 to 0.33)	0.39
	Above median baseline urate	0.35 (-0.11 to 0.80)	0.15 (-0.02 to 0.28)	0.88

Supplemental Table S6 – Relative increase in PWD split by low/high baseline urate

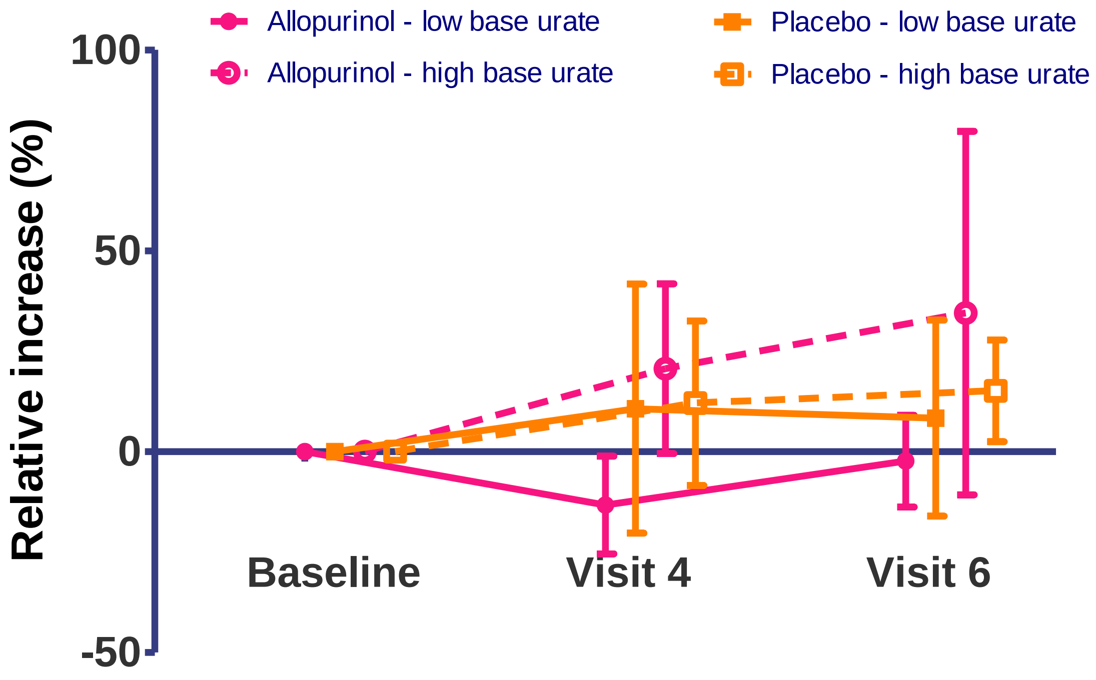

Supplemental Figure S6 – Relative increase in PWD split by low/high baseline urate

When considering Supplemental Figure S6 above, it can be seen that the lines for PWD in the allopurinol group appear to be slightly diverging, albeit with overlapping confidence intervals. To check for any difference between the low and high baseline urate sub-populations the baseline characteristics for these two groups were compared – no significant difference in any factor was found.

Also considered for those in the active treatment group was whether the <u>percentage reduction</u> in uric acid (i.e. the relative response to allopurinol) made any difference to the change in either claudication onset or peak walking distance. The median reduction in uric acid for those on allopurinol was 57.0% and this was used to split the active group in two. As can been seen in Supplemental Figure S7 and Supplemental Figure S8 below there was no significant difference between those that had a reduction in uric acid either above or below this median level.

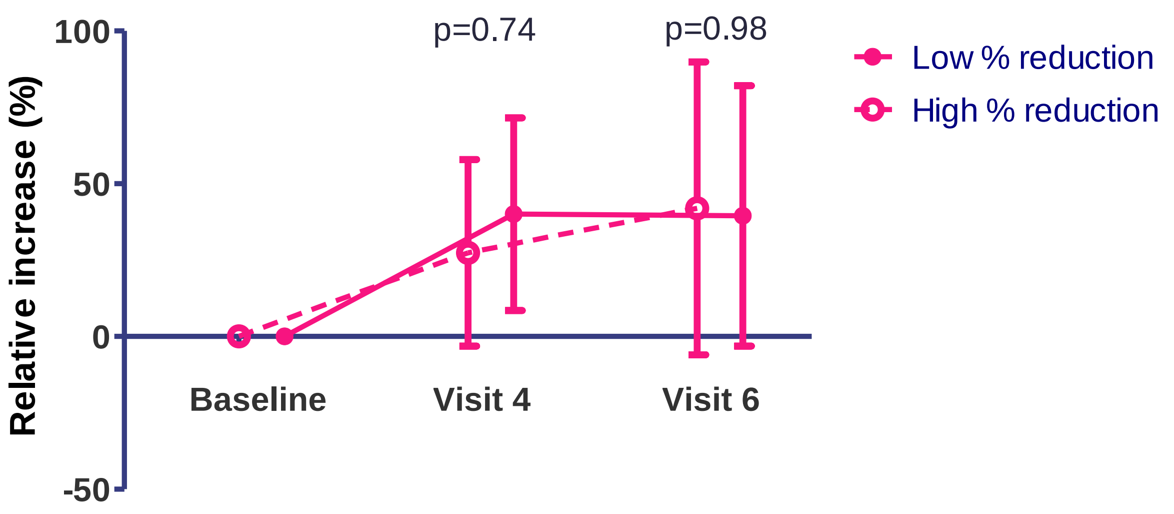

Supplemental Figure S7 – Relative increase in COD for low vs high reduction in uric acid

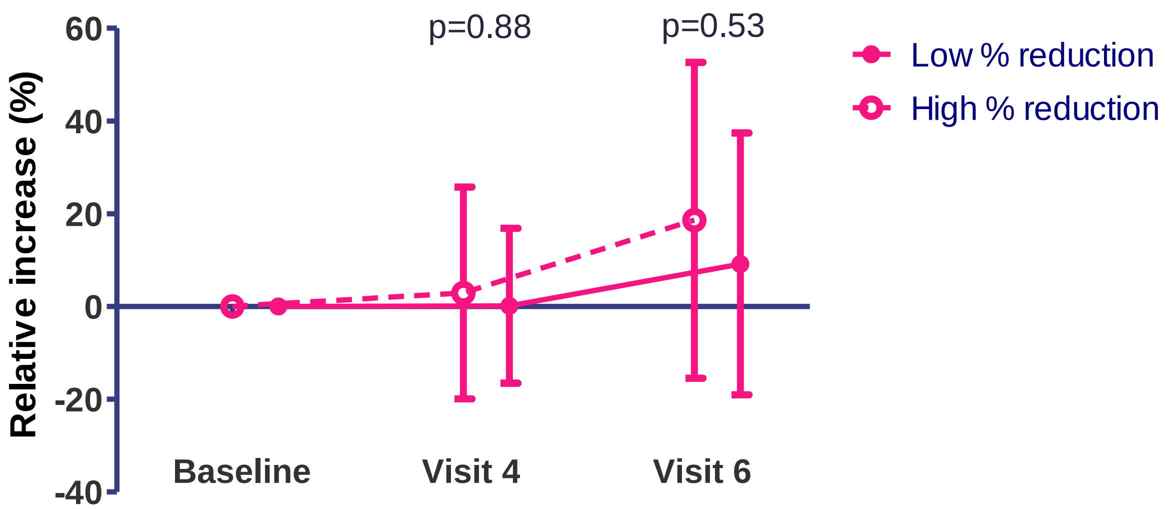

Supplemental Figure S8 – Relative increase in PWD for low vs high reduction in uric acid

1.1.4 Factor: above/below median baseline systolic BP

The median systolic BP at baseline was 154.5mmHg. The participants were split into two groups – those below and those above this median level. The relative increases in both claudication onset distance and peak walking distance were then calculated.

		Allopurinol (95% CI)	Placebo (95% CI)	p
Relative increase in COD from Baseline to Visit 4	Below median baseline systolic BP	0.32 (0.02 to 0.62)	0.28 (0.09 to 0.46)	0.95
	Above median baseline systolic BP	0.36 (0.04 to 0.68)	0.19 (-0.04 to 0.42)	0.70
Relative increase in COD from Baseline to Visit 6	Below median baseline systolic BP	0.36 (-0.10 to 0.82)	0.75 (0.39 to 1.12)	0.05
	Above median baseline systolic BP	0.45 (0.01 to 0.89)	0.25 (-0.05 to 0.56)	0.61

Supplemental Table S7 – Relative increase in COD split by low/high baseline systolic BP

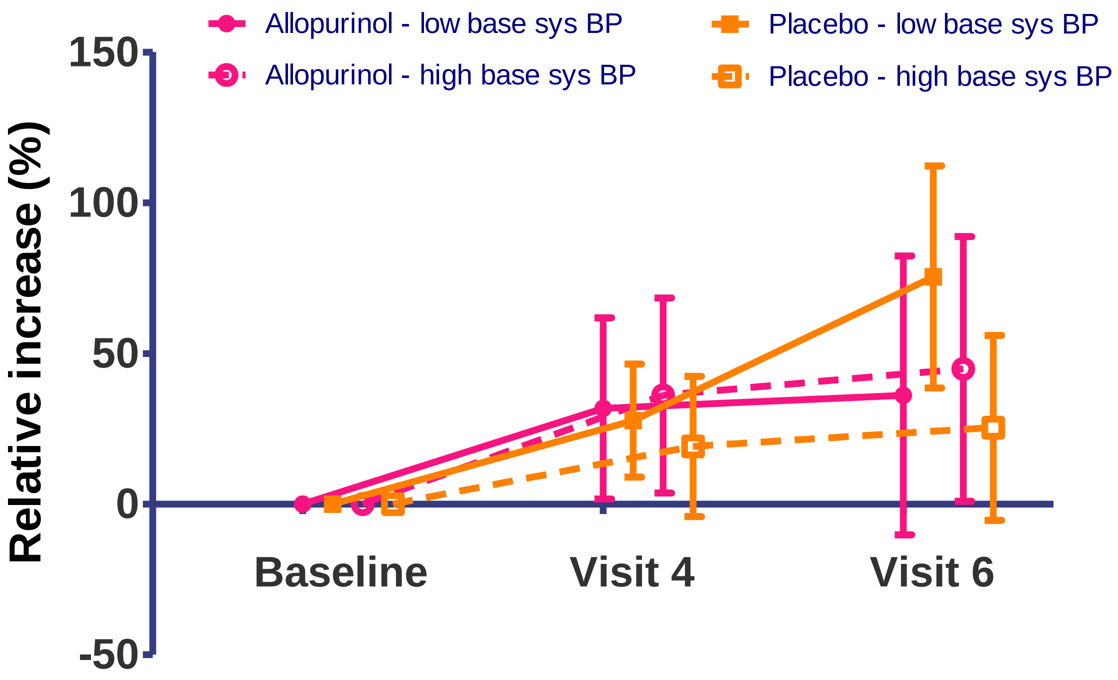

Supplemental Figure S9 – Relative increase in COD split by low/high baseline systolic BP

		Allopurinol (95% CI)	Placebo (95% CI)	p
Relative increase in PWD from Baseline to Visit 4	Below median baseline systolic BP	-0.01 (-0.12 to 0.10)	0.13 (-0.17 to 0.43)	0.52
	Above median baseline systolic BP	0.03 (-0.21 to 0.28)	0.10 (-0.07 to 0.28)	0.57
Relative increase in PWD from Baseline to Visit 6	Below median baseline systolic BP	0.04 (-0.08 to 0.16)	0.15 (-0.03 to 0.33)	0.44
	Above median baseline systolic BP	0.23 (-0.17 to 0.62)	0.10 (-0.07 to 0.27)	0.88

Supplemental Table S8 – Relative increase in PWD split by low/high baseline systolic BP

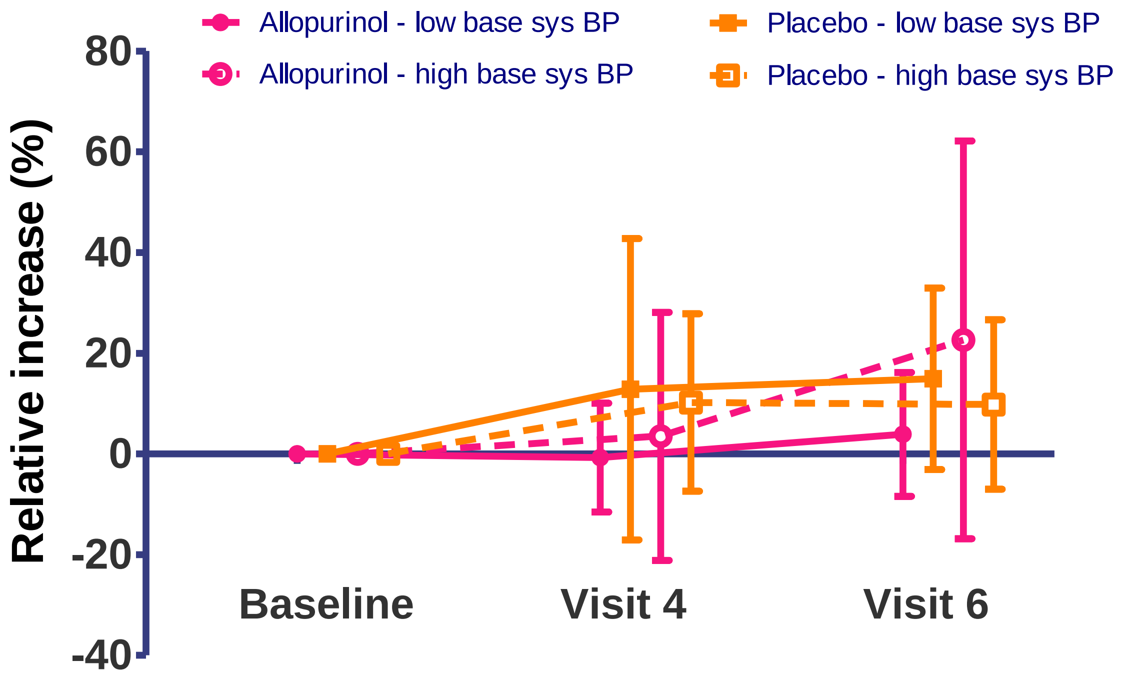

Supplemental Figure S10 – Relative increase in PWD split by low/high baseline systolic BP

1.1.5 Factor: above/below median baseline ABI

The median ABI at baseline was 0.60. The participants were split into two groups – those below and those above this median level. The relative increases in both claudication onset distance and peak walking distance were then calculated.

		Allopurinol (95% CI)	Placebo (95% CI)	p
Relative increase in COD from Baseline to Visit 4	Below median baseline ABI	0.29 (-0.02 to 0.60)	0.17 (-0.07 to 0.41)	1.00
	Above median baseline ABI	0.40 (0.08 to 0.71)	0.29 (0.10 to 0.47)	0.61
Relative increase in COD from Baseline to Visit 6	Below median baseline ABI	0.51 (-0.03 to 1.04)	0.29 (0.02 to 0.55)	0.87
	Above median baseline ABI	0.30 (0.00 to 0.60)	0.69 (0.29 to 1.08)	0.13

Supplemental Table S9 – Relative increase in COD split by low/high baseline ABI

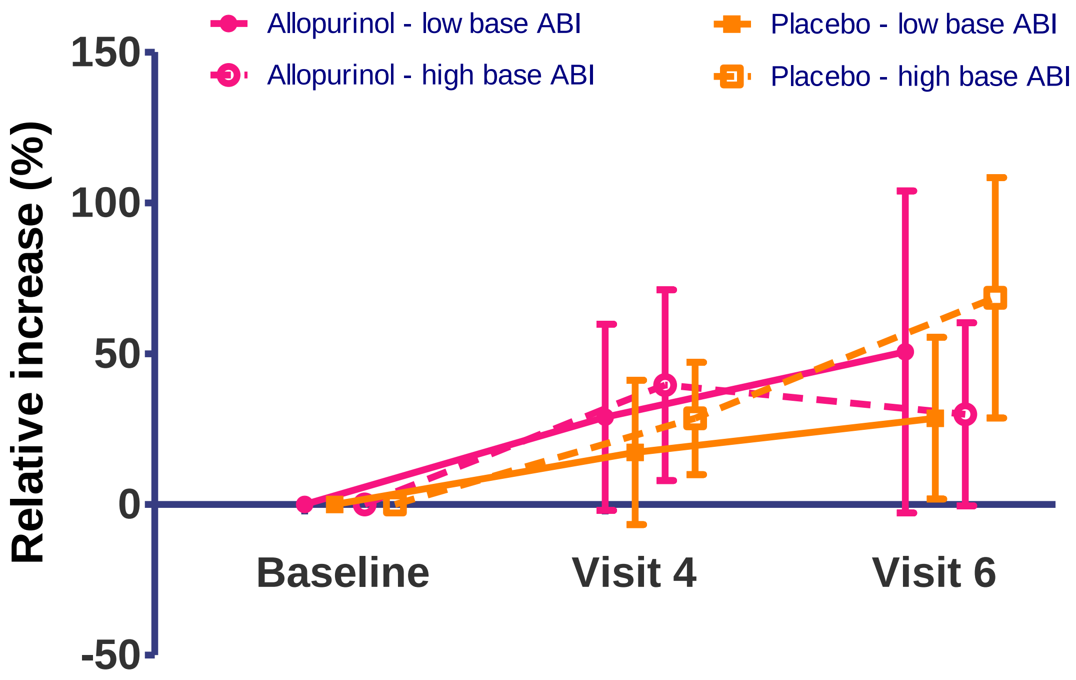

Supplemental Figure S11 – Relative increase in COD split by low/high baseline ABI

		Allopurinol (95% CI)	Placebo (95% CI)	p
Relative increase in PWD from Baseline to Visit 4	Below median baseline ABI	0.05 (-0.12 to 0.22)	0.16 (-0.16 to 0.48)	0.87
	Above median baseline ABI	-0.02 (-0.25 to 0.20)	0.08 (-0.09 to 0.25)	0.26
Relative increase in PWD from Baseline to Visit 6	Below median baseline ABI	0.15 (-0.14 to 0.43)	0.14 (-0.07 to 0.34)	0.77
	Above median baseline ABI	0.13 (-0.21 to 0.47)	0.11 (-0.04 to 0.27)	0.35

Supplemental Table S10 – Relative increase in PWD split by low/high baseline ABI

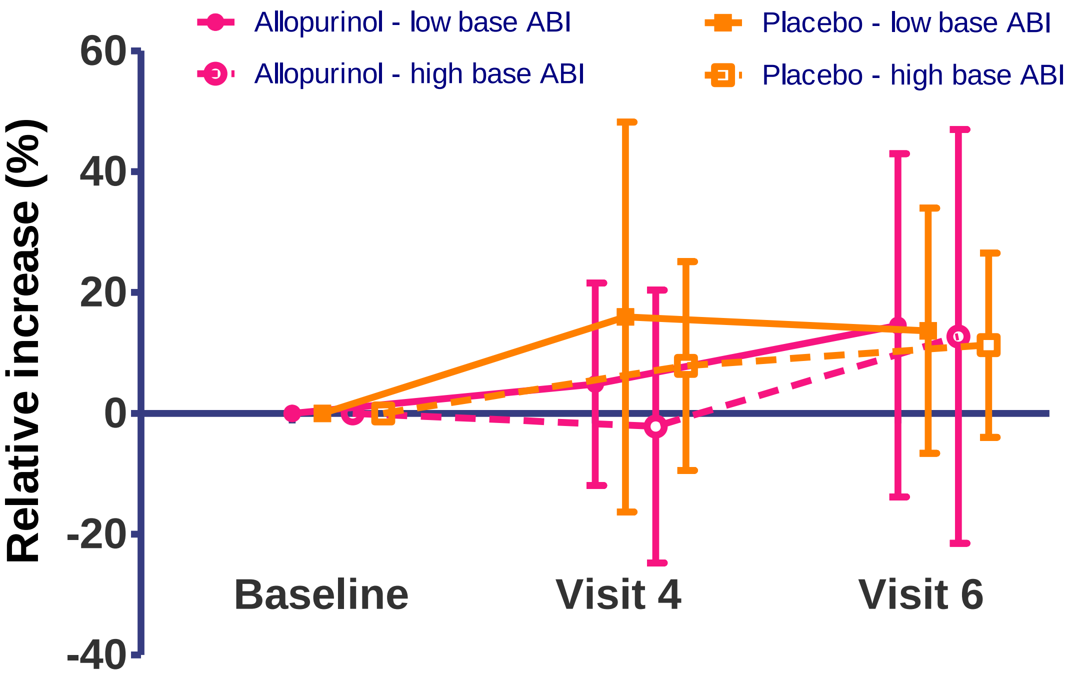

Supplemental Figure S12 – Relative increase in PWD split by low/high baseline ABI
